# First Molecular Confirmation of *Loma salmonae* in Farmed Rainbow Trout (*Oncorhynchus mykiss*) in Korea, with Histopathological Characterization

**DOI:** 10.3390/microorganisms14102260

**Published:** 2026-10-04

**Authors:** Han-Seul Cho, Hyun-Ho Song, Seung-Bin Lee, Chan Hyoek Jeon, Jeong-Ho Kim

**Affiliations:** 1 Department of Aquatic Life Medicine, Kangwon National University, Gangneung 25457, Republic of Korea; 184535@naver.com (H.-S.C.);; 2 National Fishery Product Quality Management Service, Gangneung Regional Office, Gangneung 25413, Republic of Korea; donjun@korea.kr

**Keywords:** *Loma salmonae*, microsporidia, rainbow trout, SSU rRNA

## Abstract

*Loma salmonae* is a gill-infecting microsporidian long recognized in *Oncorhynchus* species across North America, Europe, and Japan (masu/yamame salmon), but molecular confirmation in rainbow trout, or in any other salmonid host, in Korea has not previously been reported. Here, we report the first molecularly confirmed detection of *L. salmonae* infection in farmed rainbow trout (*Oncorhynchus mykiss*) in Korea, characterized by histopathology and molecular analysis, including SSU rRNA sequencing, and compared with previously characterized isolates from North America and Chile. *L. salmonae* was detected at two of five farms sampled, with an apparent prevalence of 11.0% (8/73) based on stereomicroscopic detection of gill xenomas. The detected infections were not associated with clinically apparent disease in the sampled fish; histopathology revealed limited inflammatory change and slight gill damage. SSU rRNA sequences showed 0.00% divergence from reference isolates within the 416 bp region analyzed, supporting species-level identity. Although the limited, non-random sampling of five farms in this study precludes extrapolation to estimate regional prevalence or the broader distribution of *L. salmonae* in Korean rainbow trout aquaculture, these findings establish its presence for the first time in Korea and provide a valuable foundation for broader, randomly sampled surveillance using sensitive molecular methods.

## 1. Introduction

Microsporidia are a diverse phylum of obligate intracellular, spore-forming eukaryotic parasites that infect a wide range of animal hosts, including invertebrates and vertebrates [1,2], and are now generally regarded as highly reduced fungi or fungal-related organisms based on molecular and genomic evidence [3,4]. Their most distinctive feature is the polar tube, a specialized infection apparatus used to inject sporoplasm into host cells, and their biology is further marked by extreme genome reduction and highly adapted intracellular parasitism [4,5]. Microsporidia produce environmentally resistant spores and infect hosts across a remarkably broad range of animal taxa, with individual species often showing strong host specificity [6,7,8]. In aquatic environments, they are particularly diverse among fish, crustaceans, and planktonic organisms, underscoring their ecological range and importance in aquaculture and wildlife disease systems [7,8].

Fish microsporidia constitute a diverse and biologically important group of obligate intracellular parasites that infect a wide range of freshwater, marine, and ornamental fishes, with many species exhibiting strong host and tissue specificity [7,9,10,11]. Common fish-associated genera, including *Loma*, *Pleistophora*, *Glugea*, *Pseudoloma*, *Enterospora*, *Microsporidium*, *Enterocytozoon*, and *Nucleospora*, parasitize muscle, kidney, liver, gill, intestine, and nervous tissue, producing lesions ranging from xenomas and granulomatous nodules to systemic infections. Clinical severity spans from subclinical infection to severe disease marked by chronic inflammation, growth retardation, emaciation, reduced fecundity, and mortality, depending on host species, parasite species, infection intensity, and environmental conditions [7,9,11]. In aquaculture, these infections carry considerable economic significance, reducing growth performance and market quality while increasing handling losses. The resulting diminished harvest value and elevated management costs impose a substantial economic burden, one that is further intensified under the crowded, stressful conditions typical of intensive culture systems.

Definitive diagnosis of microsporidian infection typically requires a combination of complementary methods: light microscopy and histopathology to reveal spores and the associated inflammatory response, transmission electron microscopy to resolve ultrastructural features such as the polar tube and spore wall, and molecular sequencing (e.g., SSU rRNA and other markers) for reliable species-level identification and epidemiological investigation [12]. Since microsporidian spores are highly resistant to environmental degradation, and no chemotherapeutic agents are currently approved for commercial control of microsporidiosis in food-fish aquaculture, control instead relies on preventive strategies, including biosecurity protocols, broodstock screening, sanitation, and prompt removal of infected individuals, to limit transmission within aquaculture systems [7,13]. Experimental treatments such as fumagillin and albendazole have shown efficacy in reducing xenoma numbers, though none are yet approved for commercial use [14,15,16].

Among these genera, *Loma salmonae* is a well-recognized microsporidian parasite of salmonid fishes that causes xenoma-forming infections, particularly in the gills and other epithelial tissues; however, its pathogenic significance varies considerably depending on host species, stage of infection, and culture conditions, ranging from subclinical or self-limiting infection to severe disease in susceptible hosts under intensive culture [9,14]. The parasite develops within enlarged host-derived xenomas that may contain numerous spores, and it is the rupture of these xenomas, rather than infection per se, that triggers the acute inflammatory response most closely associated with impaired respiration, reduced fish condition, and production losses [7,15]. 

*Loma salmonae* shows a broad geographical distribution, with documented occurrences in North America, as well as in Chile, Scotland, Japan, and parts of Europe [16,17]. Although the parasite is most closely associated with *Oncorhynchus* spp., particularly in marine cage aquaculture, molecular studies indicate low genetic divergence across many anadromous salmon populations, supporting effective marine transmission and suggesting that oceanic dispersal contributes to its wide distribution [17,18]. Despite this broad range, the parasite’s greatest economic impact remains concentrated in salmon aquaculture regions where susceptible hosts are cultured at high density.

The infection begins with oral entry of spores, which infect epithelial cells of the intestine before the parasite migrates to the heart, where it undergoes an approximately two-week merogony-like proliferative phase within endothelial cells [18,19]. Presporogonic stages are subsequently transported from the heart to the gill via a macrophage-mediated route, where the parasite completes sporogony within enlarged xenomas formed in gill endothelial and pillar cells [18,19]. Xenomas typically become histologically detectable four to six weeks post-exposure, and their eventual rupture releases mature spores into the vasculature, triggering an acute inflammatory response manifesting as severe, persistent proliferative branchitis. This post-rupture phase represents the most pathogenic stage of infection, with severe vasculitis contributing to clinical disease [17,18,19,20].

The severity of this process, however, is not uniform across hosts or conditions: rainbow trout tend to develop lower xenoma intensity and clear branchial xenomas more rapidly than Chinook or coho salmon, suggesting comparatively greater innate resistance to clinical disease in this host [21,22]. Expression of the infection is also strongly temperature-dependent, with xenoma development occurring within a permissive range of 9 °C to 20 °C and life-cycle progression arrested at temperatures outside this range, which has important implications for seasonal disease risk and diagnostic sensitivity in the field [23]. Because xenoma formation and spore maturation are contingent on these host- and environment-specific factors, infections can remain histologically inconspicuous or entirely subclinical under suboptimal conditions, underscoring the value of combining fluorescence-based spore visualization, histopathology, and molecular detection to avoid underestimating true infection prevalence.

In this study, we identified a microsporidian parasite infecting the gills of cultured rainbow trout (*Oncorhynchus mykiss*) in Korea during routine disease monitoring. Although no overt clinical signs were observed, xenomas indicative of microsporidian infection were detected microscopically. We visualized the microsporidian spores using a fluorescence-based stain, complemented by histopathology, and confirmed species identity through molecular sequencing and phylogenetic analysis rather than transmission electron microscopy. To our knowledge, this represents the first molecularly confirmed detection of *L. salmonae* in rainbow trout, and in any salmonid host, in Korea.

## 2. Materials and Methods

### 2.1. Fish Sampling and Observation of Parasites

**Figure 1:**
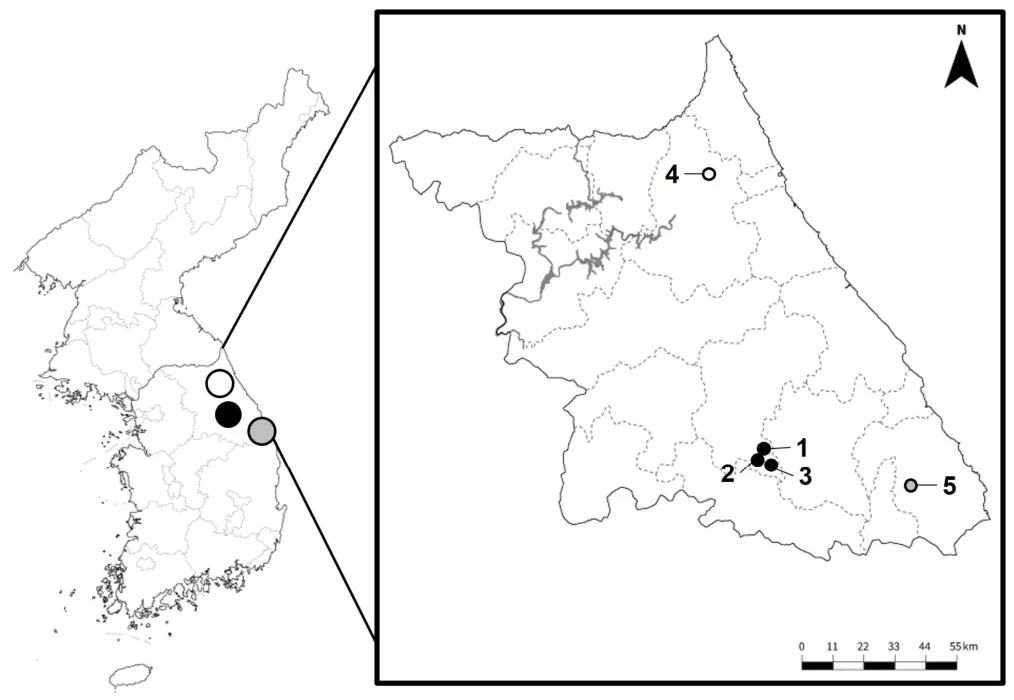
Sampling locations of the five farms across three districts (Pyeongchang, Inje, and Samcheok) in Gangwon Province, Korea. The left panel shows the general location of Gangwon Province within the Korean Peninsula; the right panel is an enlarged inset showing the precise, individually mapped locations of Farms 1–5.

We sampled rainbow trout from five commercial rainbow trout farms in Gangwon Province, on the eastern side of the Korean Peninsula, between November 2025 and April 2026 (Figure 1). Farms 1–3 were located in Pyeongchang County, while Farms 4 and 5 were located in Inje County and Samcheok, respectively; farms were selected by convenience sampling based on accessibility and farm owner cooperation. Each farm was sampled once and was confirmed, through direct communication with farm operators, to be culturing rainbow trout exclusively, with no other salmonid species present. Farms 1–3, located in Pyeongchang, drew river water from the same river, while Farms 4 and 5 each drew river water from a separate river in Inje and Samcheok, respectively; at all farms, river water was supplemented with each farm’s own groundwater as needed to maintain a stable water temperature, so the farms’ overall water supplies were not identical even where a river was shared. All fish examined were yearlings; because fish were not spawned or stocked at the same time across farms, body size varied among farm cohorts (Table 1). Within each farm, tanks/raceways were randomly selected and individual fish were collected by random netting from a single production cohort. The average water temperature ranged from 9.0 °C to 11.5 °C during the sampling periods. The number, and the average body length and weight of fish sampled at each farm are summarized in Table 1.

Sample fish were transported to the laboratory and euthanized by immersion in a bath of 2-phenoxyethanol (Sigma-Aldrich, St. Louis, MO, USA) at 0.3–0.5 mL/L dechlorinated water until opercular movement ceased and there was no response to tactile stimulation, and were left in the solution for a minimum of 10 min thereafter to ensure death; death was subsequently confirmed by an adjunctive physical method (spinal transection) prior to necropsy [24]. Following euthanasia, all fish underwent a standard gross necropsy in which the gills, liver, spleen, kidney, heart, and gastrointestinal tract were examined for gross abnormalities by naked-eye inspection followed by stereomicroscopic examination.

The gills were then dissected from the fish and placed in sterile saline. A wet mount of the gills was prepared and observed under the dissecting microscope, and if small whitish cysts (xenomas) were found, they were carefully collected. For each fish, all grossly visible xenomas were pooled and squashed with sterile saline to release spores; spore dimensions were measured from fresh wet-mount preparations of the pooled spore suspension, with individual spores randomly selected for measurement, and spores were observed under the light microscope or transferred to the fluorochrome Calcofluor White staining method for detection of chitin-containing microsporidian spores [25]. For Calcofluor White staining, a wet-mount smear of the fresh spore suspension was air-dried onto a glass slide and fixed in methanol for approximately 2 min, then stained with Calcofluor White M2R (Sigma-Aldrich, St. Louis, MO, USA) at 5 mg/mL for 5 min at room temperature in the dark, briefly rinsed with distilled water, coverslipped, and examined under a fluorescence microscope (Nikon Eclipse Ci, Tokyo, Japan) equipped with a DAPI filter set (excitation 358–365 nm, emission 445–460 nm); mature spores exhibited bright turquoise-blue fluorescence of the chitinous spore wall.

In addition, routine histopathological processing of the gills was performed. Tissue samples were fixed in 10% neutral-buffered formalin for 24–48 h at room temperature, followed by routine processing through a graded ethanol series (70%, 80%, 90%, 95%, and 100%) for dehydration, clearing in xylene, and infiltration with paraffin wax using an automated tissue processor. Processed tissues were embedded in paraffin blocks, sectioned at 4 μm thickness using a rotary microtome, and mounted on glass slides. Sections were deparaffinized in xylene and rehydrated through a descending ethanol series to distilled water, then stained with Harris’ hematoxylin for 3–5 min, differentiated in 1% acid alcohol by dipping several times, and rinsed in running tap water for 2 min. Counterstaining was performed with eosin Y for 1–2 min. Stained sections were subsequently dehydrated through ascending ethanol concentrations, cleared in xylene, and mounted with a synthetic resin mounting medium under coverslips. Slides were examined by light microscopy for histopathological evaluation [citation to be inserted]. The prevalence of infection was calculated according to the definition of Bush et al. [26] and is reported here as the apparent prevalence based on stereomicroscopic detection of gill xenomas.

### 2.2. DNA Extraction, PCR Amplification and DNA Sequencing

For each fish, all grossly visible xenomas were pooled and disrupted for DNA extraction. To disrupt the resistant microsporidian spore wall prior to DNA extraction, 200 mg of glass beads (Lysing Kit VK01, 0.1 mm diameter, Bertin Technologies, Montigny-le-Bretonneux, France) was added to the spore suspension, and spores were mechanically disrupted using a Precellys 24 tissue homogenizer (Bertin Technologies, Montigny-le-Bretonneux, France) at a speed of 6.5 m/s for 5 cycles of 30 s each, with a 2 min cooling interval on ice between cycles to prevent thermal degradation of DNA. Following mechanical disruption, spore lysis was completed by extended enzymatic digestion using the QIAamp^®^ DNA Mini Kit (Qiagen, Hilden, Germany) protocol, in which 10 μL of proteinase K was added to 190 μL of ATL buffer and incubated overnight at 56 °C, rather than the standard 1–3 h digestion period, to improve DNA yield from the resistant spore wall. DNA was eluted in a final volume of 200 µL, and DNA concentration was measured using a NanoDrop spectrophotometer (Thermo Fisher Scientific, Waltham, MA, USA) (101.6 ng/µL for strain Ls-1 and 118.7 ng/µL for strain Ls-2); 2 µL of extracted DNA was used as template per 20 µL PCR reaction. We applied 2 different primer sets for detecting microsporidian parasites collected from rainbow trout in this study; a set of LS1-LS2 primers was used for the specific detection of partial ITS region and LSU rRNA (expected amplicon size: 272 bp) of *L. salmonae* [27], and another set of CTMicrosp-Microsp1342r primers was used for the amplification of partial SSU rRNA of microsporidian parasites (expected amplicon size: approximately 1200–1300 bp, as the exact size could not be precisely calculated because the annealing position of the forward primer could not be confidently determined from reference sequences) [28], to conduct phylogenetic analysis. Detailed primer information and PCR conditions are summarized in Table 2. PCR products were electrophoresed on a 1.2% agarose gel containing EcoDye™ DNA Staining Solution (Solgent, Daejeon, Republic of Korea; 20 μL per 100 mL gel) in 1× TAE buffer at 100 V for 1 h and visualized under UV transillumination to confirm amplicons of the expected size. The PCR products were purified using an *AccuPrep*^®^ Gel Purification Kit (Bioneer, Daejeon, Republic of Korea) according to the manufacturer’s instructions. Then, 1–2 ng/μL of purified products were directly sequenced on an ABI Prism 3730xl DNA Analyzer (PE Applied Biosystems, Foster City, CA, USA).

**Figure 2:**
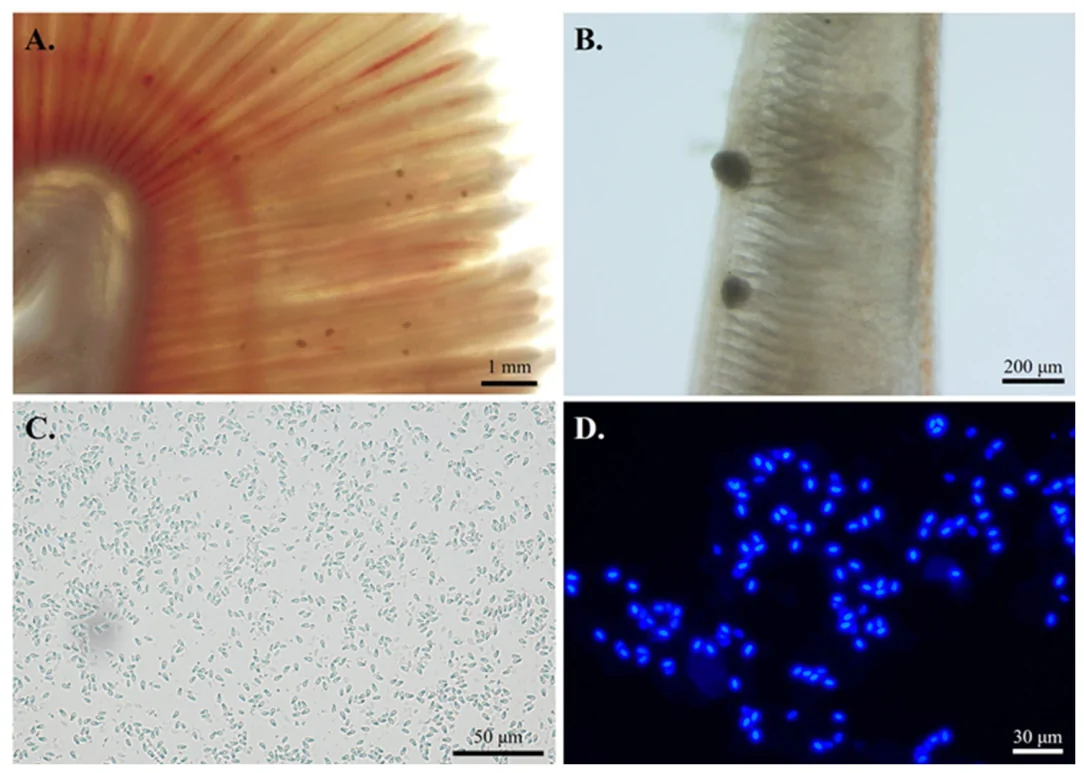
Gross and microscopic features of rainbow trout gills infected with *L. salmonae*. (**A**) Stereomicroscopic image of an excised gill arch showing multiple small, discrete, whitish-to-grayish nodules (xenomas) scattered along the gill filaments (scale bar = 1 mm). (**B**) Higher-magnification stereomicroscopic image of a gill filament bearing two round, dark grayish xenomas protruding from the lamellar surface (scale bar = 200 μm). (**C**) Fresh, unstained wet-mount preparation of spores released from a crushed xenoma, observed under light microscopy, showing numerous oval-shaped microsporidian spores (scale bar = 50 μm). (**D**) Fluorescence photomicrograph of the same spore preparation stained with Calcofluor White, showing numerous ovoid spores exhibiting bright turquoise-blue fluorescence of the chitinous spore wall under UV excitation (scale bar = 30 μm).

**Figure 3:**
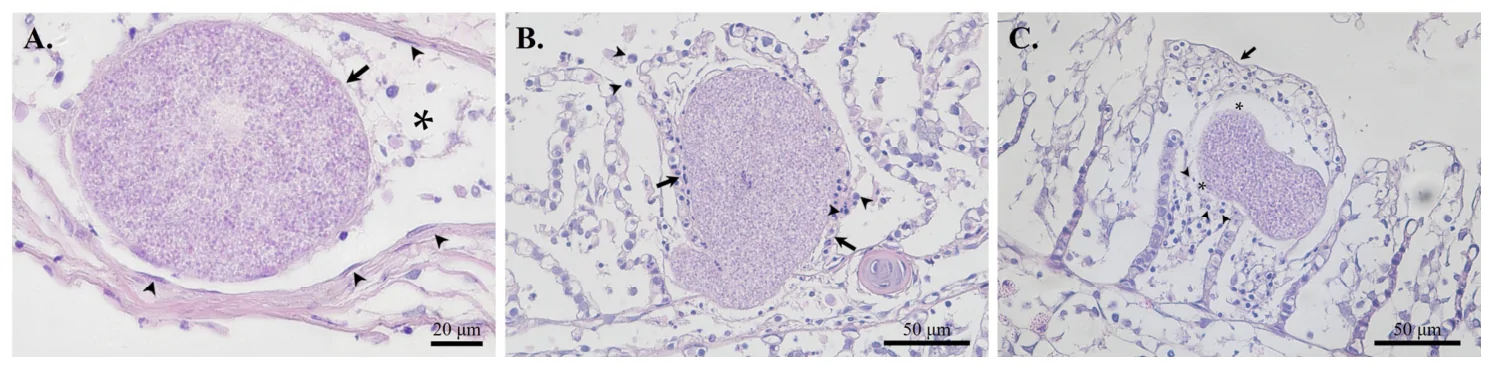
Photomicrographs of gill sections from rainbow trout infected with *L. salmonae*, H&E stain. (**A**) Higher-magnification view of a mature xenoma (arrow) occupying the lumen of a secondary lamellar capillary (asterisk), with adjacent endothelial cells (arrowheads) lining the vessel wall, illustrating the intravascular location of the xenoma within a discrete, thin-walled, round structure densely packed with numerous developing stages and mature spores; note that inflammatory cells are rarely observed. Scale bar = 20 μm. (**B**) An elongated xenoma located within the endothelial layer of a lamellar capillary, distending the vessel wall, similarly filled with developing stages and mature spores, with slight compression of the adjacent lamellar epithelium (arrows). A few inflammatory cells (arrowheads) were observed around the affected area. Scale bar = 50 μm. (**C**) Higher-magnification view of a bilobed xenoma located between secondary lamellae and surrounded by connective tissue, interpreted as an older, degenerating or spore-releasing stage based on poorly demarcated peripheral xenoma walls (upper and lower left margins) accompanied by peripheral migration of inflammatory cells (arrowheads), together with slight epithelial hyperplasia at the tip of the affected lamella (arrow). Scale bar = 50 μm.

A no-template control (nuclease-free water) was included in every PCR run for both primer sets and consistently showed no amplification. PCR using the LS1–LS2 primer pair was independently repeated twice on positive samples with consistent results. A positive control and extraction blanks were not included, as this represents the first molecular report of *L. salmonae* in Korea and no locally confirmed reference material was available; specificity was instead supported by bidirectional Sanger sequencing and BLASTn 2.17.0 analysis, which showed high identity (100% for the ITS/LSU product; 99.83–100% for the SSU product) to validated *L. salmonae* reference sequences, and by corroborating microscopic (Figure 2) and histopathological (Figure 3) findings in the same specimens.

### 2.3. The Phylogenetic Analysis

The consensus sequences were queried against the National Center for Biotechnology Information GenBank database using BLASTn 2.17.0. Species identity was assigned based on ≥99% sequence identity and high query coverage. To determine the phylogenetic placement of novel lineages, sequences were aligned with reference *L. salmonae* SSU rRNA sequences retrieved from GenBank using Clustal W [29]. Prior to tree construction, the best-fit nucleotide substitution model was selected using the Find Best DNA Models function in MEGA7 v7.0, which evaluated 24 candidate models based on the Bayesian Information Criterion (BIC); the Hasegawa–Kishino–Yano (HKY) model was identified as the best-fit model (BIC = 1879.722) and was used for the final analysis. A phylogenetic tree was reconstructed using the Maximum Likelihood algorithm in MEGA 7 [30] under the HKY model, with node support evaluated via 1000 bootstrap replicates.

## 3. Results

All examined fish showed no abnormal behavior or mortality during the sampling period, and no gross abnormalities were observed in any of the examined organs. When the excised gills were observed under a stereomicroscope, small whitish nodules suspected to be xenomas were found at various locations on the gill lamellae of rainbow trout (Figure 2A,B). We sampled five rainbow trout farms once each (*n* = 10, 13, 10, 30, and 10 fish for Farms 1–5, respectively; total *n* = 73; Table 1). Xenomas were found in fish from Farm 1 (sampled November 2025) and Farm 2 (January 2026), but not in Farms 3, 4, or 5 (Table 1). The apparent prevalence based on stereomicroscopic detection of gill xenomas was 11.0% (8/73) overall, with infections detected at two of the five farms sampled; apparently negative fish were not screened by PCR or other sensitive molecular methods. All results are summarized in Table 1. Of the eight grossly xenoma-positive fish, all eight were confirmed PCR-positive; four of these (Fish 1–4) were additionally examined histologically, and a subset of two (Fish 1 and 3) were selected for SSU rRNA sequencing, yielding strains Ls-1 (from a fish sampled at Farm 1, Pyeongchang, on 19 November 2025) and Ls-2 (from a fish sampled at Farm 2, Pyeongchang, on 20 January 2026), respectively, from two distinct individual fish. Diagnostic concordance across gross examination, histology, and PCR for each sampled fish is summarized in Table 3. The xenomas observed on the gill lamellae were nearly round, ranging from 64.0 to 184.5 μm in diameter (mean = 121.9 μm, *n* = 20). When the xenoma was crushed on a glass slide, oval-shaped microsporidian spores were found; spore dimensions, measured from fresh wet-mount preparations of pooled, randomly selected spores to avoid fixation-associated shrinkage artifacts, were 4.7 ± 0.35 μm (length) × 2.3 ± 0.18 μm (width) (range: 4.2–5.8 × 1.8–2.6 μm, *n* = 40) (Figure 2C). In addition, mature ovoid microsporidian spores exhibiting bright turquoise-blue fluorescence were observed using a DAPI-filter-equipped fluorescence microscope (Nikon Eclipse Ci microscope, Tokyo, Japan) (Figure 2D).

Histopathological examination of gill sections revealed multiple, discrete, intravascular xenomas occupying the lamellar capillaries of the secondary lamellae (Figure 3A–C). Mature xenomas appeared as round to elongated, thin-walled structures densely packed with uniform, basophilic, spherical spore/sporoblast stages, associated with limited host inflammatory infiltrate and only slight compression of the adjacent lamellar epithelium (Figure 3A,B). In contrast, some xenomas were more intensely eosinophilic and bilobed, and were accompanied by peripheral migration of inflammatory cells and slight epithelial hyperplasia at the lamellar base, all of which were interpreted as indicative of a degenerating or spore-releasing stage (Figure 3C).

**Figure 4:**
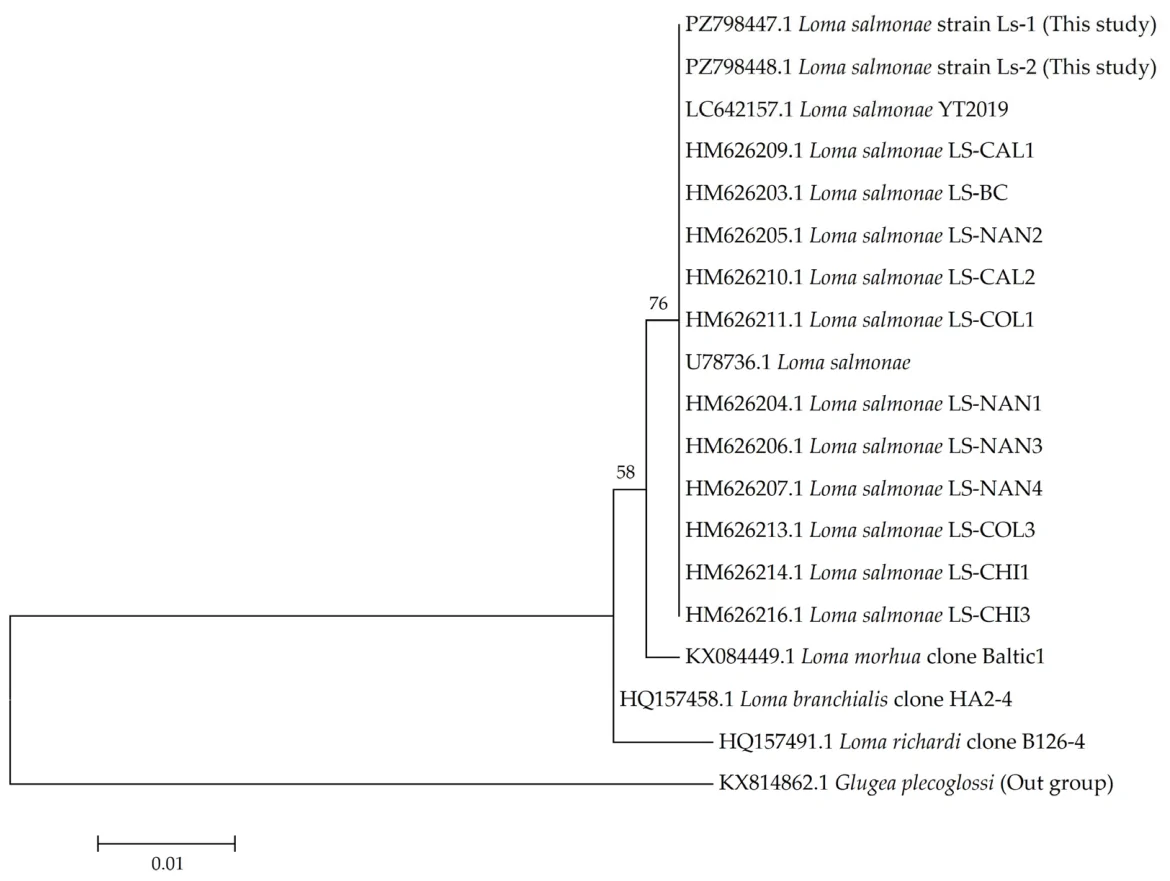
Phylogenetic tree based on SSU rRNA gene sequences showing the relationship between *L. salmonae* strains Ls-1 (PZ798447.1) and Ls-2 (PZ798448.1) obtained in this study and reference sequences of *Loma* spp. retrieved from GenBank. The 416 bp region retained after alignment and trimming represents the segment that could be unambiguously aligned without gaps across all included taxa and was used to conduct phylogenetic analysis. The tree was constructed using the maximum likelihood method based on the Hasegawa–Kishino–Yano (HKY) model, selected as the best-fit substitution model using the Bayesian Information Criterion. Numbers at the nodes indicate bootstrap support values (only values ≥50% are shown). *Glugea plecoglossi* (KX814862.1) was used as the outgroup. GenBank accession numbers precede each taxon name. The scale bar represents 0.01 substitutions per site. Tip labels retain the strain/isolate designations as submitted to GenBank by the original depositing authors; these do not represent formally defined genotypes.

A BLAST search of the GenBank database with the sequences obtained by PCR targeting the partial ITS region and LSU rDNA of *L. salmonae* (272 bp) revealed 100% sequence homology with previously registered sequences of *L. salmonae* (Table S1). Following bidirectional sequencing and quality trimming, forward and reverse reads were assembled into a single consensus sequence of 1147 bp for both strains. BLASTn analysis of this 1147 bp SSU rRNA sequence showed 100% query coverage against the closest reference sequences, with the highest pairwise identity of 99.83% to *L. salmonae* YT2019 (LC642157.1) and strain LS-BC (HM626203.1), followed by 99.74% identity to strains LS-NAN2 (HM626205.1) and LS-CAL1 (HM626209.1). For phylogenetic analysis, this 1147 bp consensus sequence was aligned with reference *Loma* spp. sequences retrieved from GenBank; because most reference sequences were themselves partial or did not overlap sufficiently across their full length, only a 416 bp region could be unambiguously aligned without gaps across all taxa and was retained for tree construction. Within this 416 bp region, our isolates grouped together with other *L. salmonae* sequences in GenBank (Figure 4). Both deposited sequences correspond to the partial SSU rRNA gene (1147 bp) amplified using the CTMicrosp–Microsp1342r primer pair. The sequences were registered in GenBank under accession numbers PZ798447.1 and PZ798448.1.

## 4. Discussion

This study provides the first molecular confirmation of *L. salmonae* in farmed rainbow trout in Korea. Gill xenomas, detected by stereomicroscopic and histopathological examination in 8 of 73 fish sampled (apparent prevalence 11.0%, based on stereomicroscopic detection of gill xenomas) across two of five farms surveyed, were morphologically consistent with the genus *Loma*, and species identity was confirmed by SSU rRNA sequencing, which showed 0.00% divergence from reference *L. salmonae* sequences within the 416 bp region analyzed. Infections were not associated with clinically apparent disease in the sampled fish. Together, these findings establish the presence of *L. salmonae* in Korean rainbow trout aquaculture for the first time.

*Loma salmonae* was first described as *Plistophora salmonae* from cultured rainbow trout in the USA [31], then transferred to a newly established genus *Loma* by Morrison and Sprague [32]. It is a common gill microsporidian of wild and captive (sea net pen cages and freshwater hatchery) salmonid fishes in both fresh and marine waters [33]. Particularly, netpen-farmed Chinook salmon (*O. tshawytscha*) has been mostly affected by *L. salmonae* in British Columbia, Canada [34]. The species was first recorded from rainbow trout in California, USA, and since then, it has appeared regularly as an epizootic in numerous species of *Oncorhynchus* in North America and Europe, possibly via translocation of contaminated eggs [33]. In Asia, Awakura et al. [35] described *Loma* sp. from masu salmon (*O. masou*) and recently Inoue et al. [36] also described *L. salmonae* from the same host species in Japan. However, no confirmed record of *L. salmonae* infection in rainbow trout, or in any other salmonid host, has previously existed in Korea. A systematic search of PubMed, Web of Science, Google Scholar, and the Korean-language databases RISS and DBpia (search terms: “*Loma salmonae*,” “*Loma* sp.,” and “microsporidia rainbow trout Korea,” in both English and Korean) identified a single prior conference abstract reporting *L. salmonae* in Korea [37]; however, this report did not describe the detection methods used, and species identity was not confirmed histopathologically, ultrastructurally, or molecularly. The present study therefore represents, to our knowledge, the first molecularly confirmed report of *L. salmonae* in Korea.

Fish microsporidia are typically transmitted directly by ingestion of environmentally resistant spores with waterborne exposure, cohabitation, and occasionally vertical transmission contributing to their persistence and impact in cultured fish populations [10]. In aquaculture systems, fish microsporidia are mainly spread via farm water systems and routine husbandry. Spores shed from infected fish enter water, sediments, and biofilms, then move between tanks, raceways, and net-pens through shared water systems and effluent, enabling horizontal transmission across the facility. Oral ingestion of contaminated feed or carcass material, cohabitation of infected and naïve stocks, and transfer of subclinically infected fish or eggs between facilities further contribute to farm-level translocation [7,13]. In South Korea, extensive disease monitoring studies have been continuously conducted across both freshwater and marine aquaculture environments to ensure the stable production and effective biosecurity management of farmed rainbow trout [38,39]. Aside from the single, methodologically unconfirmed conference abstract noted above, *L. salmonae* infection had not previously been reported despite this monitoring effort. It is not clear whether the parasite was recently introduced into Korea or was already present but overlooked, as its detection may require diagnostic approaches beyond those used in routine surveillance.

The pathogenicity of *L. salmonae* may differ depending on the affected fish species; although basically all *Oncorhynchus* species are susceptible to infection, experimental and field studies indicate that *L. salmonae* is most pathogenic in the netpen-reared Chinook salmon, causing prominent microsporidial gill disease with abundant xenomas, severe branchitis, impaired respiratory function, and substantial mortality. Rainbow trout, by contrast, are readily infected but often show minimal clinical signs and low or no mortality under experimental conditions [15,18]. As apparently negative fish in our study were not screened by PCR or other sensitive molecular methods, the true prevalence may have been underestimated, and a higher prevalence would likely be obtained using a more sensitive PCR-based detection method, which can identify infections at an early or resolving stage before xenomas become macroscopically visible. Moreover, rainbow trout is known to recover clinically from *L. salmonae* infection, with gill xenomas regressing and disappearing about 10–14 weeks post-exposure, and recovered fish develop strong protective immunity to reinfection that can last up to one year [15]. In our study, fish farmers stated that neither abnormal clinical signs nor mortality were observed in the farms during the sampling periods. The subsequent histopathological findings also showed a slight inflammatory reaction and limited structural damage to the gills. Therefore, fish farmers may not have noticed the existence of *L. salmonae* in their farms.

To further support the species-level identity of our isolate, we compared its genetic relationship with previously characterized *L. salmonae* populations. *L. salmonae* is thought to be genetically conserved across its wide host and geographic range, with very low sequence divergence; sequences of nuclear rRNA genes (SSU, LSU, and ITS) and the protein-coding EF-1α gene across wild, hatchery, farm, and laboratory isolates from North America and Chile revealed that the overall intraspecific sequence divergence is very low (generally <0.2% in rRNA and <0.03% in EF-1α), supporting recognition of a single, broadly distributed species [33]. Our two Korean isolates showed 0.00% sequence divergence from reference isolates within the 416 bp SSU rRNA region analyzed (Figure 4, Table S1), reflecting strong conservation of this region across geographically distant isolates, consistent with previous reports of low genetic divergence in this species [33]. This 0.00% divergence refers specifically to the 416 bp conserved region used for phylogenetic analysis, within which no nucleotide differences were observed among the compared isolates; this is consistent with, though numerically distinct from, the 99.74–99.83% identity obtained from BLASTn comparison of the full-length 1147 bp SSU consensus sequences against the closest GenBank references (see Section 3).

However, given the limited number of isolates examined, the short and highly conserved marker used, and the correspondingly modest bootstrap support at several nodes in the phylogenetic tree, our data do not permit inferences regarding gene flow, population structure, or the specific transmission pathways (e.g., marine dispersal) underlying this conservation; such interpretations, previously proposed in the literature for other *L. salmonae* populations [33], should be regarded as hypotheses drawn from the broader literature rather than conclusions supported by the present dataset. It should also be noted that the SSU rRNA gene is a highly conserved, single-copy marker commonly used for species-level identification, and the sequence conservation observed here reflects similarity at this specific locus only; this finding should not be interpreted as evidence of genome-wide genetic homogeneity between isolates, which would require comparison across multiple independent loci or whole-genome data. The relatively modest bootstrap support observed at several nodes in the phylogenetic tree (Figure 4) likely reflects the limited number of informative sites available within the short, highly conserved 416 bp alignment used for this analysis, rather than genuine phylogenetic uncertainty regarding species identity; low sequence variation at this locus constrains the resolving power of bootstrap analysis, particularly for nodes separating closely related isolates. Nonetheless, the clustering of our Korean isolates within the *L. salmonae* clade remained consistent, corroborating the species-level identification based on sequence conservation described above; analyses incorporating longer or more variable genetic markers would be expected to yield stronger nodal support and finer phylogenetic resolution in future studies. Indeed, pairwise divergence between *L. salmonae* and its closest congeners within this 416 bp region was consistently below 1% (Table S1: 0.48% versus *L. morhua* and *L. branchialis*; 0.97% versus *L. richardi*), indicating that this marker has limited power to discriminate among closely related *Loma* species beyond the genus level, independent of the bootstrap support limitations noted above. Broader sampling of multiple isolates from multiple host species, together with more variable molecular markers (e.g., full-length ITS or the EF-1α gene), will be necessary to clarify the genetic diversity and phylogeographic origin of *L. salmonae* in Korea.

We note that the low genetic divergence of *L. salmonae* across geographically distant isolates has previously been attributed to oceanic dispersal and marine transmission among anadromous salmon populations in marine cage aquaculture [21,33]; however, this mechanism pertains to marine and cage-based salmon culture systems rather than to freshwater rainbow trout farms such as those examined in the present study. The sequence conservation observed here is therefore better interpreted as reflecting the broader phylogenetic stability of this SSU rRNA region across the species, rather than evidence of a specific marine transmission event affecting this freshwater population; more locally relevant introduction routes for freshwater facilities are discussed below.

According to the literature, approximately 13 salmonid species (family Salmonidae) are officially recorded in Korean waters [40]. Of these, six species—specifically chum salmon (*O. keta*), Atlantic salmon (*Salmo salar*), chinook salmon, coho salmon (*O. kisutch*), and masu salmon, along with rainbow trout—have been involved in artificial propagation, commercial trials, or sea-ranching projects; however, rainbow trout, masu salmon, and Atlantic salmon account for the majority of salmonid production in South Korea [41,42,43].

All farms sampled in the present study were confirmed to culture rainbow trout exclusively, with no co-culture of other salmonid species and, accordingly, no other species available for examination; the introduction route by which *L. salmonae* entered the sampled farms could not be determined. Plausible introduction and transmission routes, including movement of infected fish or eggs, contaminated water sources or farm equipment, and the open-water river systems supplying these farms (particularly if wild salmonids or other susceptible hosts occur upstream), remain hypotheses grounded in general principles of pathogen transmission in aquaculture rather than findings of the present study, and warrant dedicated epidemiological investigation.

Beyond the farms examined here, a significant portion of salmonids undergo a freshwater juvenile phase before migrating, and *L. salmonae* is readily transmitted in both fresh and marine water across *Oncorhynchus* species [19]; this transitional stage poses a risk for pathogen transmission to other salmonid fish in facilities where multiple species are co-cultured. We therefore note, as a general consideration for the broader Korean salmonid aquaculture industry rather than as a finding of this study, that farms practicing such co-culture during the freshwater rearing phase may warrant species-inclusive disease surveillance for *L. salmonae* and other emerging pathogens, given the documented susceptibility differences among salmonid hosts described above.

Several limitations of this study should be considered when interpreting these findings. Because farms were selected by convenience rather than random sampling (see Section 2) and each was sampled once from a single production cohort, this design cannot support estimates of regional farm-level prevalence or conclusions regarding how widespread *L. salmonae* is across Korean rainbow trout aquaculture as a whole. Fish sampled from Farm 4 were also considerably smaller than those from Farms 1–3 (Table 1), consistent with the differing spawning and stocking times noted in the Methods; because the exact rearing duration at each farm could not be determined, the absence of detectable xenomas at Farm 4 may reflect a shorter window of exposure to *L. salmonae* rather than a true absence of infection, and this uncertainty likewise precludes a more detailed assessment of age-related host effects.

As noted above, molecular and histological confirmation were both restricted to grossly xenoma-positive fish (8/73 and 4/8, respectively; Table 3); fish lacking grossly visible xenomas were not examined histologically or screened molecularly, so we cannot exclude the possibility that microscopic or early-stage xenomas were present but undetected in these fish. Histological sections were examined by a single observer using qualitative descriptions (e.g., “minimal,” “mild”) rather than predefined scoring criteria, and no physiological, growth, longitudinal, or quantitative lesion assessments were performed; accordingly, the absence of clinically apparent disease at the time of sampling should not be interpreted as evidence of low pathogenicity or as ruling out disease occurrence outside the sampling period.

In summary, this study provides, to our knowledge, the first molecularly confirmed report of *Loma salmonae* infection in rainbow trout in Korea, detected at two of five farms sampled and confirmed by histopathology, fluorescence-based spore visualization, and molecular sequencing. The infections detected were not associated with clinically apparent disease in the sampled fish. Given the limited, non-random sampling design of this study, these findings should not be extrapolated to estimate regional prevalence or the broader distribution of *L. salmonae* in Korean rainbow trout aquaculture; instead, they establish its presence for the first time and raise the hypothesis that the parasite may be more widespread than previously recognized, supporting the need for broader, randomly sampled surveillance using sensitive molecular methods. Further studies incorporating a broader range of isolates and host species, together with dedicated epidemiological investigation of potential introduction and transmission pathways, will be needed to clarify the origin, genetic diversity, and epidemiological significance of *L. salmonae* in Korea.

## Figures and Tables

**Table 1:** Sampling information and apparent prevalence of *L. salmonae* infection (determined by gross observation of gill xenomas) for rainbow trout collected from five farms in Korea, including sampling date, water temperature, number of fish examined, average body length and weight, and apparent prevalence, expressed as percentage (number of infected fish/number of fish examined).

Sampling Farm	Sampling Date	Temperature (°C)	No. of Fish	Avg. Body Length (cm)	Avg. Body Weight (g)	Apparent Prevalence
Farm 1 (Pyeongchang)	2025.11.19	11.5	10	27.7	294.4	20% (2/10)
Farm 2 (Pyeongchang)	2026.01.20	11.5	13	26.6	202.1	46.2% (6/13)
Farm 3 (Pyeongchang)	2026.03.10	9.0	10	27.6	261.1	0% (0/10)
Farm 4 (Inje)	2026.04.13	10.0	30	13.5	24.3	0% (0/30)
Farm 5 (Samcheok)	2026.04.22	9.5	10	31.5	325.1	0% (0/10)
**Total**			**73**			**11.0% (8/73)**

**Table 2:** Oligonucleotide primer sets and PCR conditions.

Primers	Sequences (5′-3′)	PCR Conditions	References
LS1	CTGGATCAGACCGATTTATAT	95 °C for 30 s, 46 °C for 30 s, 72 °C for 1 min, 35 cycles	[27]
LS2	ATGACATCTCACATAATTGTG		
CTMicrosp	CACCAGGTTGATTCTGCCTGACG	95 °C for 30 s, 58.7 °C for 30 s, 72 °C for 1 min, 35 cycles	[28]
Microsp1342r	ACGGGCGGTGTGTACAAAGAACAG		

**Table 3:** Diagnostic concordance across gross examination, histology, PCR, and sequencing for the 8 gill xenoma-positive rainbow trout identified in this study.

Fish ID	Gross Xenoma	Histology	PCR	Sequenced
1	+	+	+	Ls-1
2	+	+	+	—
3	+	+	+	Ls-2
4	+	+	+	—
5	+	—	+	—
6	+	—	+	—
7	+	—	+	—
8	+	—	+	—

+, positive; —, not examined histologically (Histology column) or not selected for sequencing (Sequenced column); not a confirmed negative result.

## Data Availability

The data supporting the findings of this study are available in this article and its Supplementary Materials. Raw data supporting the findings of this study are available from the corresponding author, upon reasonable request.

## Supplementary Materials

The following supporting information can be downloaded at https://www.mdpi.com/article/10.3390/microorganisms14102260/s1. Table S1: Pairwise genetic distances (%) among *L. salmonae* strains Ls-1 and Ls-2 (this study) and reference *Loma* spp. sequences from GenBank, based on a 416 bp SSU rRNA alignment (*Glugea plecoglossi* as outgroup; GenBank accessions precede taxon names). The 0% divergence among *L. salmonae* sequences reflects conservation within this fragment only and should not be read as evidence of genome-wide homogeneity.

## Abbreviations

The following abbreviations are used in this manuscript:

BLAST	Basic local alignment search tool
DAPI	4′,6-diamidino-2-phenylindole
EF-1	Elongation factor-1
H&E	Hematoxylin and Eosin
ITS	Internal transcribed spacer
LSU	Large subunit
PCR	Polymerase chain reaction
SSU rRNA	Small subunit ribosomal RNA
